# The Sichuan Mental Health Survey: Methodology

**DOI:** 10.3389/fpsyt.2021.645355

**Published:** 2021-09-17

**Authors:** Rong-cheng Su, Li-hong Huang, Jia Li, Bo Zhou, Jia-jun Zhao, Hui Li

**Affiliations:** ^1^Key Laboratory of Psychosomatic Medicine, Chinese Academy of Medical Sciences, Chengdu, China; ^2^Sichuan Provincial Center for Mental Health, Sichuan Academy of Medical Sciences and Sichuan Provincial People's Hospital, Chengdu, China

**Keywords:** epidemiologic research design, survey mode, fieldwork, survey sampling, sample weights

## Abstract

The Sichuan Mental Health Survey (SMHS) is a provincially representative survey with a coherent methodology to obtain the prevalence of multiple mental disorders and data of services used and to analyze the psychological and social risk factors or correlates in Sichuan, China. Mental disorders include anxiety disorders, mood disorders, schizophrenia, and other psychotic disorders, drug use and alcohol use disorders, impulse control disorder, and eating disorders. A cross-sectional design is employed to sample adults from 200 communities/villages in all 21 prefectural-level municipalities of Sichuan Province in a five-stage provincially representative disproportionate stratified sampling design. The participants need to be interviewed face to face by trained interviewers from local primary healthcare institutions and by psychiatrists. The quality control staff implement data quality control by checking records and statistics in the interview system, and then re-interviewing checks are done by the psychiatrists. Data is weighted to adjust the sample distribution to match the whole population. The outcomes of the SMHS would not only demonstrate the serious challenges posed by the high burdens of mental disorders but also offer baseline data for policymakers and healthcare professionals to study and resolve the factors that influence mental health in Sichuan, China.

## Introduction

In the past 40 years, thanks to the Reform and Opening Up Policy, Sichuan Province, along with China, has undergone extraordinary social change and economic development. This transition has led to huge changes in demographics, urbanization, economic prosperity, migration, transportation, education, culture, social concepts, environmental pressure, and disease epidemiology. These changes may have negative impacts on the prevalence and disease burden of mental disorders. According to a systematic review, the disease burden caused by multiple mental disorders has accounted for about 13% of all non-communicable disease burden in China (1). So, mental health has become a crucial public health and social problem that has to be monitored by Chinese policymakers and healthcare professionals. In order to improve the mental health of citizens, the Chinese and Sichuan provincial governments have issued a series of regulations and policies. Moreover, the upcoming mental health promotion part of the “Healthy China Initiative 2030” will also focus on improving the mental health system of China.

Although the mental health system of China continues to develop, it remains to have a huge number of untreated people with mental illness. More specifically, its efficiency is plagued by the high prevalence of mental disorders and low consultation rates (2, 3), the high proportion of refractory mental disorders and poor medications responses (4, 5) as well as insufficient and unevenly distributed mental health resources (6–8). The intense stigma associated with mental illness, a lack of mental health professionals and specialists, and the culturally specific expressions of mental illness may play a role in the disparity.

The results of earlier surveys (2, 9–13) all reflect some magnitude of the increasing prevalence of mental disorders in several parts of China. However, the generalizability of these results is limited by disparities in the definitions and measurements of disorders as well as a lack of representativeness. As for the usage of mental health services, the Chinese Health Service Surveys (14–16) implemented in recent decades mainly focused on chronic diseases; little focus has been put on the mental health services used by the public (17, 18). Drastic changes have occurred in the treatment of mental disorders over the past decades, but a provincially representative survey that mainly focuses on the usage of the services of mental disorders in Sichuan is still missing.

To resolve the methodological limitations of previous studies, an epidemiological survey, using the revised methodology of China Mental Health Survey (CMHS) (19), with consistent diagnostic classification, fully structured diagnostic questionnaires, and comprehensive household survey technic (20, 21), for Sichuan province, is crucial to acquire representative data of mental disorders and service usage in Sichuan, China. The three major objectives of the SMHS are to investigate the prevalence of multiple mental disorders, to obtain data about mental health service usage of people with mental disorders, and to study the risk factors or correlates of mental disorders and mental health services from social and psychological aspects. The SMHS, designed and organized by the Sichuan Provincial Center for Mental Health (SPCMH), could obtain the extent of the problem and provide background information for the improvement of the mental health system that can sufficiently meet the mental healthcare needs of the entire population of Sichuan province.

## Survey Mode

The SMHS is the first provincial survey using the cellphone-based WeChat Mini Program Interview (WMPI) mode for data collection, carried out by trained and qualified interviewers in the field of psychiatric epidemiology, which is more portable and feasible than computer-assisted personal interview (CAPI) mode (22). The WeChat mini-programs are “sub-applications” within the WeChat ecosystem that can provide advanced features to users, such as e-commerce, task management, coupons, *etc*. An interview system, a centrally controlled mini-program, is developed to streamline the field investigation and ensure quality. In China, the majority of psychiatric epidemiological surveys, such as the two large-scale Chinese epidemiological surveys of mental disorders in 1982 (23) and 1993 (24), adopt paper-and-pencil interview (PAPI) mode due to limited access to information technology and cellphones/computers. PAPI can be carried out simply by printing out questionnaires, which is more practical in fieldwork, but extra data entry is needed and it is without timely quality control (22, 25). Furthermore, the Structured Clinical Interview for DSM-IV-TR Axis I Disorders, Research Version, Patient Edition (SCID-I/P) is quite a lengthy questionnaire with complicated skip rules. So, interviews could be carried out much easier by using WMPI mode. Furthermore, PAPI could be less effective because of the high expenses for printing, transferring, and storing, with potentially extra costs and mistakes in data entry. Finally, quality control by WMPI could be more timely and credible because of the availability to analyze the paradata, administrative data about the survey like how many times there are contacts with each interviewee, of the interviews.

The decision of not implementing telephone, mail, or Internet method is made not only because of several methodological concerns, such as avoiding low response rate and even imposture (22), but also because no cellphone network or Internet is available in some remotely mountainous areas in Sichuan, China. Due to the fact that the SMHS interview includes some sensitive questions, such as illegal drug use, sex life, suicide, and domestic violence, audio computer-assisted self-administered interviewing (ACASI) could be a potential option for increasing report rates on sensitive questions (26–29), but considering the unsatisfied concordance (30, 31) and extra technical assistance (32) needed among the low-educated population, ACASI mode is not applied. Therefore, WMPI mode is selected for SMHS.

Based on WMPI mode, a dedicated interview system for the survey is developed, which is made up of several modules containing sample allocation and replacement, interview schedule and reminder, built-in questionnaires, data transfer and storage, quality control, and feedback. In the remote areas with limited access to the Internet, the survey can be administrated in PAPI mode and entered into the system afterward.

### The Survey Population

The SMHS aims to have a provincially representative sample among Chinese residents aged 18 years and over while living in the local community for at least 6 months. In consideration of communication difficulties, residents with hearing loss are not included in the sampling population.

### Sampling

#### Sampling Design

The interviewees are selected from a five-stage provincially representative multi-stage disproportionate stratified sampling design using demographic data offered by the Municipal Institute of Mental Health (MIMH) in all 21 prefectural-level cities of Sichuan Province.

The first stage of the sampling is to select 25 districts and counties from all 21 prefectural-level municipalities of Sichuan province, which are divided into six regions based on location, economy, culture, and ethnicity, using the sampling method of probability proportionate to size (PPS). Considering the number of districts and counties in each region and the total population of residents, the number of districts and counties in each region is proportional to the square root of the population.

The second stage is to select streets or towns from the 25 counties or districts. Four streets or towns are selected from each county or district using the method of PPS, which was based on the total number of permanent residents living in each street or town, totaling to 100 streets or towns.

The third stage is to select communities or villages from the 100 streets or towns. During this process, two communities or villages from each street or town are selected, totaling to 200 communities or villages.

To alleviate the pressure on the primary healthcare institutions to collect detailed demographic information of residents, the data collection of stages four and five is split into two stages in SMHS, instead of only one stage in CMHS, resulting in only 110% sample size of demographic information having been collected instead of the whole population.

The fourth stage is to select households from communities or villages. Firstly, all 21 MIMHs complete the coding of households in all 200 sampled communities or villages, with the help of local primary healthcare institutions. Then, systematic sampling is applied to select specific households. The number of households to be sampled in each community/village is calculated by multiplying the proportion of the population of each community/village to the total population of all 200 communities/villages by the total sample size; 10% is added as reserves for sample replacement. Vacant household, commercial tenant, invalid address, one household with multiple houses, and multiple households in one address are verified. If more than one financially independent household is living at the same address, one household is randomly selected. If not enough households are found, the supplement can be made from neighboring communities/villages.

The fifth stage is to select respondents from the household samples by using Kish table sampling (33). Firstly, all 21 MIMHs complete the registration of accessible residents in the sampled households of all the 200 sampled communities or villages, with the help of local primary healthcare institutions. Accessible household members are sorted by sex (male and female) from youngest to eldest, adjusted due to the fact that the Kish table is developed during the post-World War II baby boom in the USA. Then, one respondent from every sampled household is selected, using the randomly prescribed order of the eight Kish sampling tables.

#### Sampling Size

According to the survey objectives of the SMHS and the results of CMHS, the sample size is calculated by the mental disorder with the lowest prevalence, which is the lifetime prevalence of schizophrenia or any other psychotic disorder at 0.7% according to the results of CMHS. On top of our experiences in this field and budget limits, the target sample size is determined by assuming that the prevalence (*p*) for mental disorders would be 1%. To achieve a relative error (ε) of 15%, the following formula (34) is used:


$$\begin{array}{l} n=\frac{Z_{1-\frac{\alpha }{2}}^{2}\left(1-p\right)}{p\varepsilon^{2}} \end{array}$$

It would require the sample size to be 16,910 individuals. Considering the problems commonly encountered in the survey, such as denial of the interview, relocation, household registration errors, difficulty in establishing contact with respondents, etc., the final sample size is set to 20,300 individuals, with an increase of 20%.

#### Sample Replacement (Stage I Survey Only)

To ensure that at least 80% of the sample size is completed and the finished samples have a similar distribution to the general population, sample replacement, as a coordination and quality control method, could happen during the sampling and the formal investigation. Sample replacement during the sampling—only one chance is given to verify the accessibility of sampled respondents after the fifth stage—is not counted in the replacement rate. However, sample replacement during the formal investigation, including telephone appointment and field investigation, is counted in the replacement rate, set at 10%, so as to avoid the interviewers from replacing hard respondents with easy ones. Sample replacement can also reduce the need for interviewers to manually enter the interview records for replaced respondents afterward since the interview system has 10% reserves, thereby improving the efficiency and speeding up the survey progress. Telephone follow-up for the replaced respondents is carried out by third-party outsourced call center service providers, and the reasons of non-response are recorded for further quality control.

#### Replacement Procedures

The replacement request is applied by the interviewers in the interview system and approved by the MIMHs. Then, the system will randomly select respondents from the 10% reserves within the same community/village.

Finally, each community/village should finish no <80% of its sample size, as designed in the sample size calculation. If not finished, its superior Municipal Health Commission is criticized by the Provincial Health Commission, and special supervisions would be carried out by the SPCMH.

#### Sample Weighting

Since the SMHS applies a multi-stage disproportionate stratified sampling design, four procedures are implemented to get the weighted prevalence, including sampling design weights, item non-response adjustment weights, post-stratification adjustment weights, and trimming of the weights.

#### Sampling Design Weight

The sampling design weight is the inverse sampling probability of a sample being included through all the sampling stages in the sample design; this includes first-stage county or district sampling weights, second-stage town or street sampling weights, third-stage village or community sampling weights, fourth-stage households coding/address sampling weights, and fifth-stage respondent sampling weights. The final sampling design weight is calculated by multiplying the above-mentioned five weights.

#### Item Non-Response Adjustment Weights

Non-response could happen at both the unit level and the item level. With sample replacement in place, unit non-response, existing in most stages of the sampling procedure, is controlled. Then, the imputation method (35) is used to adjust for item non-response adjustment.

#### Post-Stratification Adjustment Weights

In sampling surveys, certain important indicators cannot be obtained before sampling, resulting in structural biases of the sample, so post-stratification adjustment weight is used to compensate for the sampling errors and improve the precision of target variable estimation.

In the epidemiological survey of mental disorders, gender, age, and urban or rural areas are very important indicators (22, 36) and are used in the SMHS to create the post-stratification adjustment weight.

#### Trimming of the Weights

The complexity of the sample design, the item non-response, and the post-stratification adjustment cause a great deviation in weights and affect the efficiency of target variable estimation. So, the mean square error (MSE) is controlled by trimming the weights to a certain range.

Nonetheless, the trimming could affect the effectiveness of a predesigned post-stratification adjustment weight. So, several attempts of post-stratification adjustments along with the trimmings are carried out to ensure both the MSE and the effectiveness of the post-stratification adjustment.

Based on literature review (22, 37, 38) and experiences in the public mental health field, the cutoff points of the trimmings are set at 0.01 and 0.99 quantile of post-stratification adjustment weights.

### Fieldwork Procedures and Organization

The SMHS is designed to be implemented in two stages. Stage I survey is conducted by trained interviewers, and stage II survey is finished by psychiatrists. Both stages are supervised by the Department of Public Health, SPCMH, along with the MIMHs in all 21 prefectural-level cities of Sichuan Province.

Two project managers from the SPCMH are in charge of the stage I and II surveys. The project managers are responsible for internal and external coordination during fieldwork implementation, system design, quality control, and data management.

In the stage I survey, 277 persons, including 42 members from quality control teams and 235 interviewers, join in data collection. All interviewers are recruited from local counties or communities to avoid difficulties in different accents or dialects and also to cut expenses on accommodation and travel. During the field investigation, each sampled survey district or county is managed and supervised by the corresponding MIMH. At the beginning of the project, supervisors supervise the work progress of the interviewers through telephone, WeChat, QQ, SMS platform, and email and provide quality feedback, responses to question, safety tips, bill reimbursement, remuneration application, and equipment recycling. In the later stage of the investigation, the supervisors lead the team to tackle key problems in areas with difficulties in interviews and slow progress, ensuring that the sample size will be met. The group leaders are nominated among the interviewers and are mainly responsible for the communication with the interviewers and interviewees, in addition to the local authorities, including the health services center or neighborhood committee, and to arrange for household surveys. The interviewees are allocated by the supervisors, whose information is inputted into the system in advance, and the interviewers log in the system to obtain samples and conduct interviews.

A letter explaining the aims and significance of the study is given to the interviewee before the interview starts, and the interviews could be carried out only if written informed consent is given. A card containing the telephone number 96111, the Provincially Mental Assistance Hotline of Sichuan Province, is also given for further assistance and inquiries. One gift package valued at 30 Yuan is given to the respondent as a reward for participation and reimbursement for time. Up to three attempts for non-contact households or interviewees and two visits to those who refused to be interviewed are required to raise the response rate. During this process, sending different interviewers is also implemented; especially second visits to those who refused to be interviewed are done by psychiatrists.

In the stage II survey, 121 psychiatrists recruited by all 21 MIMHs in Sichuan Province carry out interviews under the supervision of the quality control teams. In consideration of regional culture, dialect, and habit similarity with the respondents, stage I interviewers could help introduce the psychiatrists to the respondents. The psychiatrists are blinded to the screening results of psychoses or dementia before giving the diagnoses of schizophrenia and other psychotic disorders or dementia. Written informed consent is also needed before the clinical interview. A gift or cash valued at 50 Yuan is given to the respondents for the finished interview.

According to the Mental Health Law of China, diagnoses of mental disorders can only be made by psychiatrists in a registered hospital. Therefore, the results of SCID would not be given to community-interviewed respondents. Help cards with information on local mental health facilities are instead provided to the respondents and their relatives if the person is thought to have a suicide plan or has attempted suicide recently. They are advised to see psychiatrists in these facilities.

The recruitment, consent form, and field procedures are approved by the institutional review boards of the SPCMH and all 21 MIMHs.

### Fieldwork Quality Control

Comprehensive and responsive quality control of fieldwork is the cornerstone of the SMHS. As mentioned in the sample design, the sample selection of households is implemented by the SPCMH centrally, and the respondents are selected using Kish sampling tables that are inserted into the system, which could avoid the preference of interviewers for easier samples. The WMPI mode, equipped with skip logic, made it possible to reduce misuses of the SCID-I/P by skipping the useless questions automatically and avoiding item non-response. Questionnaire data and paradata are uploaded to the system to be analyzed by the quality control team on a daily basis, implementing data check (100%), audio record check (20%), telephone check (10%), and field check (1%). The oral agreements of audio recording from the respondents should be obtained and recorded. Paradata mainly consists of the intermediate and final results code of each interview, such as the time of interview for each question, the number of contact efforts, the contact information of the respondents and corresponding rural/urban doctors, addresses of respondents, and audio recordings of the interviews.

A total of 46 quality control staff from SPCMH and MIMHs carry out the *post-hoc* checks; all have taken the training organized by the SPCMH, including stages I and II interview training course, with extra training on quality control procedures. Data check is implemented to identify structural deficiencies of the questionnaire data and to detect interviews with unreasonable length, under-reporting behaviors by analyzing the distribution of interview data and paradata on a daily basis. Audio record check is mainly used to assess the proper use of the scales, using computer-assisted web interviewing system. In the first 3 days of fieldwork, one randomly selected interview of each interviewer is checked each day, and timely feedback is provided, and later checks are implemented proportionately among interviews. Telephone check is carried out to find replaced interviews or fake interviews and verify several key issues using computer-assisted telephone interviewing system. Field check is done to visit some households and respondents along with the stage I interviewers by quality control staff and stage II psychiatrists so as to give timely instruction. Emphasis is made on timely responses during the whole process of quality control. Should problems be detected, the interviewer would be reached and instructed to re-contact the respondent for the missing data. In some cases, the interview may have to be implemented by another interviewer again.

### Payment for Interviewers

The scales for the stage I survey are relatively brief, with no stem–branch logic questions and with a minimal variation in the length of the interview, so the interviewers are paid by the hour, not the interview, to allow for more patience and carefulness.

Payment by the interview applies in the stage II survey, with a minimal variation in the length of the SCID-I/P interview and more trust being given to professional psychiatrists.

### Interviewer Training

The SPCMH organizes the 1-day stage I survey training course for all 398 survey participants, including 42 members from quality control teams and 235 interviewers, which contains epidemiological survey methodology, techniques, and communication skills in field investigations, as well as stage I questionnaire scales. All qualified interviewers should have passed performance-based certificate interviews and knowledge-based quizzes. A continuous 4-day stage II survey training course is held for 42 members from quality control teams and 121 psychiatrists on using SCID-I/P and Mini-Mental State Exam (MMSE) scales. All qualified psychiatrists should pass the final exam and reliability test. The interview system training is also provided.

### Value

Rich information about mental disorders, risk factors or correlates, and services use from the perspective of services demanders is collected in this survey. Researchers from multiple disciplines can carry out an analysis of the abundant data so as to enrich our understanding and knowledge of mental health in Sichuan, China. The epidemiological data will also be of remarkable value for mental health system reforms and further experimental and analytical studies, thus reducing the disease burden of multiple mental disorders in Sichuan, China.

### Differences With CMHS

Firstly, the local primary healthcare institutions of Sichuan province are at the disposal of SPCMH, authorized by the Health Commission of Sichuan Province, the provincial health administration who issued a dedicated official document to its subordinate administrative agencies requesting to deploy human, material, and financial resources to assist in the on-site interview. This is completely different with CMHS, which is more of a scientific survey project, lacking support from the local health bureau. With experienced staff from local primary healthcare institutions recruited as stage I interviewers in the SMHS, the respondents are more receptive than the lay interviewers of CMHS, which may improve the authenticity and reliability of responses.

Secondly, in the fourth stage, households from communities or villages were selected. The CMHS identifies several residential groups consisting of 50 households in each community or village, and one residential group is randomly selected among them. Then, 28–50 households are selected according to the estimated response rate in each community or village, based on previous survey experiences and considerations of the nature and location of the community or village. However, this is totally different in SMHS, which has a strict reason classification for non-response respondents to implement quality control and randomly replaces respondents with the pre-reserved 10% samples. So, the demographic distribution of completed survey respondents is more similar to the general population than that of CMHS.

Thirdly, a huge amount of human, material, and financial resources is being put into the field investigation. To ensure authenticity and reliability, coordination and quality control work must be strictly controlled. With limits of a centrally controlled 10% sample replacement reserves and 80% sample size being finished, a delicate balance between administrative management and academic requirements is achieved by the SMHS.

Finally, the SMHS is the first provincial survey to use the cellphone-based and centrally controlled WMPI system for data collection, carried out by trained and qualified interviewers in the field of psychiatric epidemiology, which is more portable and feasible than the CAPI mode of CMHS, with real-time positioning and recording function to ensure timely coordination and quality control.

## Overview

The Sichuan Mental Health Survey is the first provincially representative survey of mental disorders and mental health services among adults in Sichuan, China. The SMHS is carried out by face-to-face interviews using the WMPI interview system. A two-step procedure is applied for mental disorders, schizophrenia, and other psychotic disorders, using GHQ-12 scales as the screening instrument and SCID-I/P (39) as the diagnostic tool. Dementia is screened by AD8 scales and diagnosed by the MMSE scales in a two-step design. The detailed measurements used for the SMHS will be described in a separate article. This article mainly presents the overview of the survey design and field procedures of the SMHS.

## Data Availability Statement

The original contributions presented in the study are included in the article/supplementary material, further inquiries can be directed to the corresponding author/s.

## Ethics Statement

The studies involving human participants were reviewed and approved by Ethics Committee of Sichuan Academy of Medical Sciences and Sichuan Provincial People's Hospital. The patients/participants provided their written informed consent to participate in this study.

## Author Contributions

R-cS: designed and implemented the project. L-hH: in charge of quality control of scales. JL: in charge of fieldwork implementation. All authors contributed to the article and approved the submitted version.

## Funding

This study was supported by the Health Commission of Sichuan Province, the provincial health administration. This study was also supported by grants from the Science and Technology Bureau of Chengdu (2019-YF09-00099-SN).

## Conflict of Interest

The authors declare that the research was conducted in the absence of any commercial or financial relationships that could be construed as a potential conflict of interest.

## Publisher's Note

All claims expressed in this article are solely those of the authors and do not necessarily represent those of their affiliated organizations, or those of the publisher, the editors and the reviewers. Any product that may be evaluated in this article, or claim that may be made by its manufacturer, is not guaranteed or endorsed by the publisher.
